# Alteration of protein expression pattern of vascular endothelial growth factor (VEGF) from soluble to cell-associated isoform during tumourigenesis

**DOI:** 10.1186/1471-2407-5-128

**Published:** 2005-10-04

**Authors:** Ratchada Cressey, Onusa Wattananupong, Nirush Lertprasertsuke, Usanee Vinitketkumnuen

**Affiliations:** 1 Department of Associated Medical Science, Chiang Mai University, Chiang Mai, Thailand; 2 Department of Pathology, Chiang Mai University, Chiang Mai, Thailand; 3 Department of Biochemistry, Chiang Mai University, Chiang Mai, Thailand

## Abstract

**Background:**

Vascular endothelial growth factor (VEGF) is a potent mitogen for endothelial cells, and its expression has been correlated with increased tumour angiogenesis. Although numerous publications dealing with the measurement of circulating VEGF for diagnostic and therapeutic monitoring have been published, the relationship between the production of tissue VEGF and its concentration in blood is still unclear. The aims of this study were to determine: 1) The expression pattern of VEGF isoforms at the protein level in colorectal and lung adenocarcinoma in comparison to the pattern in corresponding adjacent normal tissues 2) The relationship between the expression pattern of VEGF and total level of circulating VEGF in the blood to clarify whether the results of measuring circulating VEGF can be used to predict VEGF expression in tumour tissues.

**Methods:**

Ninety-four tissue samples were obtained from patients, 76 colorectal tumour tissues and 18 lung tumour tissues. VEGF protein expression pattern and total circulating VEGF were examined using western blot and capture ELISA, respectively.

**Results:**

Three major protein bands were predominately detected in tumour samples with an apparent molecular mass under reducing conditions of 18, 23 and 26 kDa. The 18 kDa VEGF protein was expressed equally in both normal and colorectal tumour tissues and predominately expressed in normal tissues of lung, whereas the 23 and 26 kDa protein was only detected at higher levels in tumour tissues. The 18, 23 and 26 kDa proteins are believed to represent the VEGF_121_, the VEGF_165 _and the VEGF_189_, respectively. There was a significant correlation of the expression of VEGF_165 _with a smaller tumour size maximum diameter <5 cm (p < 0.05), and there was a significant correlation of VEGF_189 _with advanced clinical stage of colorectal tumours. The measurement of total circulating VEGF in serum revealed that cancer patients significantly (p < 0.001) possessed a higher level of circulating VEGF (1081 ± 652 pg/ml in colorectal and 1,251 ± 568 pg/ml in lung) than a healthy volunteer group (543 ± 344 pg/ml). No correlation between the level of circulating VEGF and the pathologic features of tumours was observed.

**Conclusion:**

Our findings indicate that the expression patterns of VEGF isoforms are altered during tumourigenesis as certain isoform overexpression in tumour tissues correlated with tumour progression indicating their important role in tumour development. However, measurement of VEGF in the circulation as a prognostic marker needs to be carefully evaluated as the cell-associated isoform (VEGF_189_), but not the soluble isoform (VEGF_121 _and VEGF_165_) appears to play important role in tumour progression.

## Background

VEGF plays a crucial role in tumour expansion by initiating permeabilization of blood vessels, by extravasation of plasma proteins, by invasion of stromal cells, and by causing the sprouting of new blood vessels that supply the tumour with oxygen and nutrients [1]. As a result of alternative splicing, 6 VEGF isoforms of 121, 145, 165, 183, 189 and 206 amino acids are produced from a single gene [2]. Due to differential incorporation of basic residues encoded by exon 6 and 7, VEGF isoforms differ in their heparin-binding properties, membrane association, and secretion [3]. VEGF_121_, which lacks the basic residues of both exons, does not bind heparin-containing cell surface proteoglycan [4], and is freely soluble. VEGF_165 _is also secreted. However, cationic residues in exon 7 enable VEGF_165 _to bind heparin, thus, some remains bound to the cell surface or to extracellular matrix. VEGF_189 _which retain both exons, has the highest affinity for heparin and therefore, remains tightly cell associated.

Detection of circulating VEGF has been investigated as a potential serum diagnostic marker for malignant disease and for inflammation [5]. Increased serum concentrations of free VEGF have been measured in various types of cancer, including brain, lung, gastrointestinal, hepatobiliary, renal, and ovarian cancers [6]. However, the relationship between the pattern of the production of VEGF protein isoforms in tumour tissues and their concentration in the circulation is still unclear.

A number of studies have shown that expression of certain VEGF transcripts are correlated with tumour progression. Increased mRNA expression of VEGF_189 _is correlated with poor prognosis in osteosarcoma [7] and non-small cell lung cancer [8,9], whereas expression of VEGF_121 _was correlated with lymph node metastasis in primary lung tumours [10]. Although increases of certain VEGF transcripts have been demonstrated to correlate with the progression of various tumours, the actual protein levels of the different VEGF isoforms and their significance during cellular transformation are unknown. Moreover, it has been suggested that elevated protein expression in tumour tissues was mediated by both enhanced transcription [11] and translation [12]. Thus, in order to understand the role of VEGF in tumour progression, it is important to investigate expression of different VEGF isoforms at the protein level during tumourigenesis. To our knowledge, no studies focusing on the VEGF isoform pattern at the protein level and their relationship with respect to total VEGF in the circulation have been reported.

Therefore, the aims of this study were to determine: 1) The protein expression pattern of VEGF isoforms in colorectal and lung tumours in comparison to the corresponding adjacent normal tissues in order to understand whether specific VEGF protein isoforms play an important role during tumourigenesis. 2) The relationship between the expression pattern of VEGF and the level of total circulating VEGF in the blood.

## Methods

### Selection of patients and sample

Between April 2002 and June 2004, samples were collected from cancer patients at Maharaj Nakorn Chiang Mai Hospital, which comprised 76 colorectal tumours (averaged age was 59 ± 15.2 (mean ± SD), 46 females and 30 males) and 18 non-small cell lung tumours (averaged age was 55 ± 14.6, 10 females and 8 males, 9 adenocarcinomas and 9 squamous cell carcinomas). In each case, adjacent normal tissue was collected. These specimens were immediately placed in vials, frozen in embedded medium for the preservation of cell integrity, and stored at -80°C until analyzed. Samples were graded by a pathologist according to the pathological features of the tumours, which included tumour size in maximal diameter, histological grading, lymph node metastasis, distant metastasis, and tumour staging (the AJCC TNM classification).

To avoid pre-analytical sample-to-sample variation due to blood collecting procedures, each blood sample was allowed to clot for at least 4 hrs before collecting serum as it has been reported that the release of VEGF during clotting period would have reached a plateau by this time [13]. Of 94 patients recruited in this study, serums were obtained from 56 cancer patients prior to the operation (38 from colorectal cancer patients, 18 from lung cancer patients). The age range of cancer patients was 58 ± 12.5 years and composed of 32 females and 24 males.

Serums were also collected from 47 healthy volunteers with no history of rheumatoid arthritis or recent pregnancy, trauma, surgery (within 1 month) or menstruation (within 1 week) using the same procedure as for the cancer patients so that a comparison could be made. The age range of healthy volunteers was 51 ± 10.9 (mean ± SD) years, composed of 20 female and 27 males. All serums were stored at -70°C until analyzed. The study was approved by the ethical committee of the Faculty of Medicine, Chiang Mai University (document number 56/2545).

### Western blotting

Western blotting was performed to evaluate the expression of VEGF in each tissue. Frozen tissues were thawed, cut into small pieces and homogenized in SDS lysis buffer (0.5 M Tris-HCl pH 6.8, 2% SDS (w/v) and 10% glycerol (v/v)) containing a protease inhibitors cocktail (104 mM AEBSF, 0.08 mM aprotinin, 2.2 mM leupeptin, 3.6 mM bestatin, 1.5 mM pepstatin A, 1.4 mM E-64; Sigma, U.S.A). The tissue homogenate was then centrifuged at 10,000 g for 15 minutes at 4°C, after which the supernatant was removed and the protein concentration of the supernatant was estimated using the BCA protein assay kit (PIERCE, U.S.A). Twenty-five micrograms of protein from the tumour tissue and normal tissue from each patient was resolved on a 10% SDS polyacrylamide gel under reducing conditions and electrotransferred onto a nitrocellulose membrane (Biorad, U.S.A). After blocking with 5% non-fat milk in TBS containing 0.05% Tween-20 (TBS-Tween) for 1 hour, the membrane was incubated with anti-VEGF antibodies (Santa Cruz Biotechnology, Inc., USA, Cat. no. SC-152, dilution 1:1000) for 1 hour. After washing with TBS-Tween, the membrane was incubated for 1 hour at RT with horseradish peroxidase-conjugated goat anti-mouse IgG (Dako, U.S.A). After washing with TBS-Tween, immunoreactive protein was visualized with a chemiluminescence-based procedure using the ECL Plus detection kit according to the manufacturer's protocol (Amersham, U.S.A). In order to examine the equality of protein loaded, the amount of total protein loaded into each lane was examined by staining with coomassie blue.

### Measurement of total VEGF in serum

For the detection of circulating VEGF in serum, enzyme-linked immunosorbent assay (ELISA) was performed using two different anti-VEGF antibodies purchased from R&D system, USA. Briefly, capture antibodies specific for VEGF (R&D System, cat no. AF293 at concentration 200 ng/ml) was immobilized onto-96-well microtiter plates. Unbound antibody was removed by washing the plate and a blocking reagent was added. Following a wash, recombinant VEGF protein standard (VEGF_165_) diluted in PBS containing 5% BSA to various concentrations (75–2,500 pg/ml), unknown serum and control serum were then incubated with the solid phase antibodies, which capture VEGF. After washing away unbound molecules, a detection antibody specific for VEGF (R&D System, cat no. MAB293 at concentration 500 ng/ml) was added. After incubation and washing, HRP-conjugated anti-mouse immunoglobulin was added. The plate was washed and a TMB substrate solution (Zymed, USA) was added. After 20 minutes, the color development was stopped and the intensity of color was measured using a microtiter plate reader (450 nm). The color developed in proportion to the amount of bound VEGF. When we measured 20 serum samples twice in two separated assays, the inter-assay variation ranged between 5–10% within the same concentration range. The average recovery of the added recombinant VEGF_165 _ranged between 85–115%, indicating an acceptable level of specificity of the assay.

### Statistical analysis

Total VEGF levels are expressed as mean ± standard deviation. Differences in the circulating VEGF level of two independent groups were evaluated using the Mann-Whitney test. Correlation between VEGF isoform expression and the pathological features were evaluated using chi-square test. All the statistical evaluations were performed by using the SPSS for Window version 10.0 (SPSS, Inc., Chicago, IL, USA).

## Results

### Pattern of VEGF protein expression in normal and tumour tissues of colon and lung

**Figure 1:**
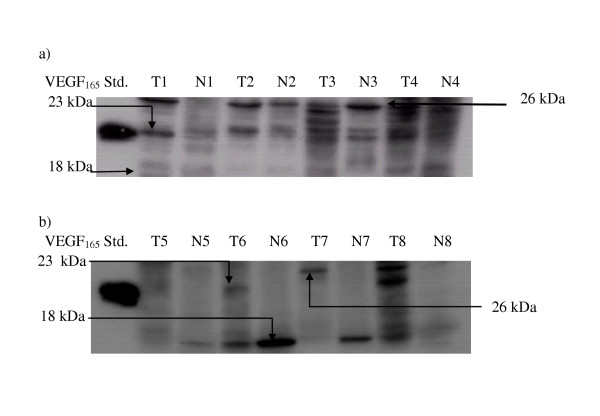
Representative Western blots showing protein expression pattern of VEGF isoform in (a) colorectal and (b) non-small cell lung tumour tissues and their corresponding adjacent normal tissues (T, tumor tissues; N, normal tissues).

The expression pattern of VEGF isoforms in tumour tissues in comparison to normal tissues determined by western blot analysis are shown in Figure 1. Three major protein bands were predominately detected in colorectal and lung tumour samples with an apparent molecular mass under reducing conditions of 18 kDa, 23 kDa, and 26 kDa. The 18 kDa VEGF was equally expressed in both normal and tumour tissues of colorectal and predominately expressed in normal tissue of lung, whereas the 23 and 26 kDa were only detected at higher levels in tumour tissues of both organs. Expression of the 23 kDa VEGF isoform was observed in 55.3% (42 of 76 patients) of colorectal tumours and 88.9% (16 of 18 patients) of lung tumour tissues. Whereas, expression of 26 kDa VEGF isoform was detected in 69.7% (53 of 76 patients) and 88.9% (16 of 18 patients) of colorectal and lung tumour tissues, respectively.

### Protein expression patterns of VEGF isoforms in tumour tissues of colon and lung in relation to pathological features

The two types of cancer were classified according to the pathologic features, which included tumour sizes in maximum diameter, depth of invasion, lymph node metastasis, distant metastasis and histological differentiation. Expression of the VEGF isoforms in relation to the pathological features of colorectal tumours and lung tumours are summarized in Table 1.

**Table 1:** Summary of relationship between VEGF isoform expression and pathologic features in colorectal and non-small cell lung cancers (p < 0.05 was considered significant)

**Pathologic features**	**VEGF isoform (kDa)**			
	
	VEGF 23 kDa	*p *value^a^	VEGF 26 kDa	*p *value^a^
**Colorectal cancer (total 76 cases)**	42 (55.3%)		53 (69.7%)	
**Gender**				
Female (46 cases)	28 (60.8%)	0.224	32 (69.5%)	0.968
Male (30 cases)	14 (46.7%)		21 (70.0%)	
**Tumour size**				
≤ 5 cm (45 cases)	30 (66.7%)^b^	< 0.05	30 (66.7%)	0.483
> 5 cm (31 cases)	12 (38.7%)		23 (74.1%)	
**Histological differentiation**				
Well (42 cases)	22 (52.4%)	0.574	28 (66.7%)	0.517
Moderate or Poor (34 cases)	20 (58.8%)		25 (73.5%)	
**Tumour stage grouping**				
Early stage (I or II) (34 cases)	23 (67.6%)	0.09	19 (55.9%)	< 0.01
Late stage (III or IV) (42 cases)	19 (45.2%)		34 (80.9%)	
**Metastasis**				
No (49 cases)	31 (63.3%)	0.059	29 (59.2%)	< 0.01
Yes (27 cases)	11 (40.7%)		24 (88.9%)	
				
**Lung cancer (total 18 cases)**	16 (88.9%)		16 (88.9%)	
**Gender**				
Female (10 cases)	8 (80.0%)	0.477	8 (80.0%)	0.477
Male (8 cases)	8 (100.0%)		8(100%)	
**Tumour size**				
≤ 5 cm (7 cases)	7 (100.0%)	0.231	7 (100.0%)	0.231
> 5 cm (11 cases)	9 (81.8%)		9 (81.8%)	
**Histological differentiation**				
Well (5 cases)	4 (80.0%)	0.490	4 (80.0%)	0.490
Moderate or Poor (13 cases)	12 (92.3%)		12 (93.3%)	
**Tumour stage grouping**				
Early stage (I or II) (2 cases)	1 (50.0%)	0.210	0 (0.0%)	< 0.01
Late stage (III or IV) (16 cases)	15 (93.8%)		16 (100%)	
**Metastasis**				
No (7 cases)	6 (85.7%)	1.00	5 (71.4%)	0.137
Yes (11 cases)	10 (90.9%)		11 (100.9%)	

^a ^chi-square test, ^b ^percentage in relation to total number of cases in each pathological features

No significant difference between gender of the VEGF expression pattern was observed in both types of cancer. In colorectal cancer, it was found that expression of VEGF isoform with molecular weight 23 kDa was significantly correlated with a smaller tumour size (maximum diameter < 5 cm, p < 0.05), whereas the 26 kDa VEGF isoform was significantly correlated with advanced clinical stage and metastasis of the tumour (p < 0.01). Expression of the 26 kDa VEGF isoform was also significantly correlated with advanced clinical stage of non-small cell lung cancer (p < 0.001). Sixteen (out of 18) lung tumour tissues which overexpressed 26 kDa VEGF were late stage tumours (Table 1). No significant difference of the expression pattern of VEGF between different histology type (adenocarcinoma and squamous cell carcinoma) was observed (data not shown).

### Levels of circulating VEGF in cancer patients compared to healthy volunteers and their relationship to pathological features

**Figure 2:**
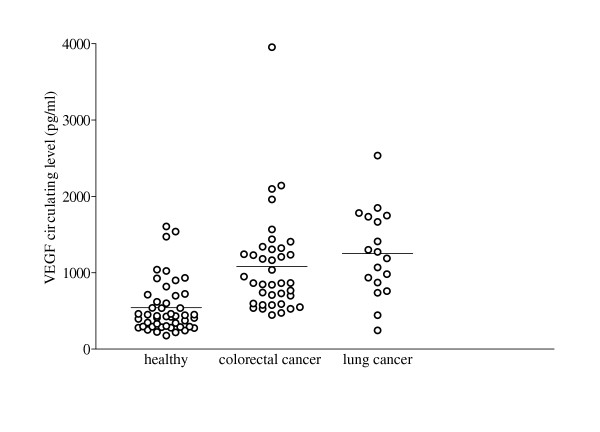
Serum level of circulating VEGF of colorectal and non-small cell lung cancer patients in comparison to healthy volunteers.

Preoperative serum was collected from 56 cancer patients; these included 38 patients with colorectal cancer and 18 with lung cancer. Serum from 47 healthy volunteers was also collected for comparison. The result showed that cancer patients possessed significantly higher level of circulating VEGF than those in healthy volunteers (Figure 2). While level of total circulating VEGF in healthy volunteer was only 543 ± 344 pg/ml, it was 1081 ± 652 pg/ml and 1,251 ± 568 pg/ml in patients with colorectal and lung cancer, respectively (Table 2). No significant relationship between the level of circulating VEGF and the pathological features was observed (Table 3). Gender also did not show any impact on circulating level of VEGF (Table 3). In addition, none of the VEGF isoforms showed a significant relationship with the serum level of VEGF. Although colorectal cancer patients with overexpression of VEGF 23 kDa, which is believed to be VEGF_165 _(one of a secretable form of VEGF) in tumour tissues, possessed higher levels of circulating VEGF in serum (1190 ± 752 pg/ml) than those possessing undetectable level of VEGF_165 _(875 ± 330 pg/ml), it was not statistically significant (p = 0.207, Mann-Whitney test).

**Table 2:** Serum level of VEGF in colorectal and non-small cell lung cancer patients in comparison to healthy volunteers (p < 0.05 was considered significant)

**Type of sample**	**No. of cases**	**VEGF concentration (pg/ml)**	***p *value**^**a**^
Colorectal cancer patients	38	1,081 ± 652^b^	<0.001
Lung cancer patients	18	1,251 ± 568	<0.001
Healthy volunteers	47	543 ± 344	

^a^Mann-Whitney test, ^b^mean ± SD

**Table 3:** Serum level of VEGF in relation to the clinicopathologic features of colorectal and non-small cell lung cancers (p < 0.05 was considered significant)

**Pathological features**	**No. of cases**	**VEGF concentration (pg/ml)**	***p *value**^**a**^
**Colorectal tumours**	38	1,081 ± 652^b^	
**Gender**			
Female	22	987 ± 470	0.433
Male	16	1212 ± 841	
**Tumour size**			
≤ 5 cm	23	1134 ± 727	0.411
> 5 cm	15	1002 ± 531	
**Tumour stage grouping**			
Early stage	23	1166 ± 799	0.777
Late stage	15	954 ± 303	
**Metastasis**			
No	27	1132 ± 749	0.910
Yes	11	961 ± 304	
**VEGF 23 kDa**			
Positive	25	1190 ± 752	0.207
Negative	13	875 ± 330	
**VEGF 26 kDa**			
Positive	26	1125 ± 720	0.742
Negative	12	989 ± 486	

**Lung tumours**	18	1,251 ± 568	
**Gender**			
Female	10	1297 ± 509	0.374
Male	8	1194 ± 666	
**Tumour size**			
≤ 5 cm	7	1304 ± 661	0.762
> 5 cm	11	1217 ± 532	
**Tumour stage grouping**			
Early stage	2	716 ± 666	0.206
Late stage	16	1318 ± 541	
**Metastasis**			
Yes	8	1125 ± 655	0.556
No	10	1331 ± 522	
**VEGF 23 kDa**			
Positive	16	1190 ± 574	0.160
Negative	2	1741 ± 9.8	
**VEGF 26 kDa**			
Positive	16	1224 ± 590	0.482
Negative	2	1468 ± 395	

^a ^Mann-Whitney test, ^b^mean ± SD

## Discussion

It has become clear that the growth of solid tumours is dependent on the process of angiogenesis and that VEGF is a central positive regulator of this process. Most VEGF-producing cells appear preferentially to express VEGF_121_, VEGF_165 _and VEGF_189_. In this study, we investigated the expression pattern of the VEGF protein isoform in colorectal tumour tissues and in lung tumour tissues and compared them with the expression pattern of normal tissues from each organ, respectively. Three major protein bands with molecular weight 18, 23 and 26 kDa were predominately detected. The 23-kDa protein band is believed to be the VEGF_165 _as this band was at the same position as the human recombinant VEGF_165 _protein standard (R&D System, USA) used in this study. Expression of VEGF_145 _and VEGF_206 _is comparatively rare seemingly restricted to cells of placental origin [14,15]. Therefore, protein bands with molecular weight of 18 and 26 kDa are assumed to be VEGF_121 _[16,17] and VEGF_189_, respectively.

In colorectal tumours, it was found that VEGF_121 _was expressed equally in both tumour and normal tissues, whereas the VEGF_165 _and VEGF_189 _were only detected at higher level in tumour tissues. However in lung tumour, VEGF_121 _appeared to be predominately expressed in normal tissues, whereas VEGF_165 _and VEGF_189 _were predominately expressed in tumours tissues. Protein expression of VEGF_165 _correlated significantly with a smaller tumour size, whereas VEGF_189 _correlated significantly with advanced clinical stage and metastasis of the tumours. Although only 18 lung tumours were investigated in this study, the 26-kDa VEGF isoform was also overexpressed significantly in advanced stage of the tumour (Table 1).

Although the regulation of VEGF expression is becoming well understood, its mode of action, particularly the regulation of expression and distribution of the three primary isoform (VEGF_121_, VEGF_165 _and VEGF_189_), remains unclear. It has been demonstrated that overexpression of smaller isoforms resulted in hemorrhagic events, but the expression of VEGF_189 _resulted in increased vessel density [18]. In this study we found that overexpression of VEGF_189 _protein isoform, but not VEGF_121 _or VEGF_165_, was associated with advanced tumour stage. Our data is consistent with the previous reports where expression of VEGF_189 _transcripts was correlated with poor prognosis in non-small cell lung, osteosarcoma, renal, colorectal and esophageal cancer [7-9,19,20]. It was also suggested that up-regulation of VEGF_189 _might result in increased angiogenesis, tumour growth and metastasis in a colon cancer cell line [21]. Moreover, VEGF_189 _has been demonstrated to be a potent permeability factor in vivo [22], supporting the role of this isoform in the control of angiogenesis.

VEGF_165 _has also been demonstrated to play an important role in tumourigenesis. When different isoforms of VEGF were transfected into the VEGF-null cells in isolation and the transfected cells were implanted into nude mice, it was found that VEGF_165 _was the most prominent isoform that can fully rescue expansion of the angiogenesis-deficient tumour, while VEGF_121 _and VEGF_189 _only partially or failed completely to rescue tumour growth, respectively [23]. However, these authors suggested that VEGF isoforms work in a coordinated fashion to recruit and expand tumour vasculature. In our study we found that although VEGF_165 _was predominately expressed in colorectal tumour tissues, its expression was significantly correlated with smaller tumour size (maximum diameter less than 5 cm.). Although expression of VEGF_121 _mRNA has been previously reported to be correlated with lymph node metastasis [10] of primary lung cancer and the invasiveness of bladder cancer [24], in our study we found that level of the 18 kDa VEGF protein, which believed to be VEGF_121 _[16], was equally expressed in both normal and tumour tissues of colorectal, and predominately expressed in normal tissues of the lung.

Detection of VEGF has long been known as a potential serum diagnostic marker for malignant diseases. Increased serum VEGF concentrations have been measured in various types of cancer, including, brain, lung, gastrointestinal, hepatobiliary, renal and ovarian cancer [25]. However, the relationship between the pattern of the production of VEGF protein isoforms in tumours and its concentration in the circulation is still unclear. In this study, we determined the expression pattern of VEGF isoforms in tumour tissues in relation to the level of total VEGF in a patient's serum. The comparison of the VEGF level in serum of cancer patients with that of normal volunteers revealed that cancer patients possessed significantly (p < 0.001) higher levels of VEGF in serum. However, some normal volunteers also possessed quite a high level of VEGF, which may due to the possibility that normal tissues, like lung tissue (Figure 1) can also produce VEGF_121 _that is secretable into the circulation. In addition, no significant relationship between level of circulating VEGF and pathologic features was observed.

## Conclusion

Our findings indicate that the expression patterns of VEGF isoforms are altered during tumourigenesis as certain isoform overexpression in tumour tissues correlated with tumour progression indicating their important role in tumour development. However, measurement of circulating VEGF in serum may have limited use as a tumour marker. This may be due to the following reasons: 1) The VEGF isoform that appeared to be significantly correlated with tumour progression is VEGF_189_, which is the cell-associated isoform, is not soluble. 2) Some normal tissues, i.e. lung (as shown in Figure 1), expressed high-level VEGF isoforms (VEGF_121_) that secreted into the circulation. 3) Expression of some secretable VEGF isoforms (VEGF_165_) was negatively correlated with the progression of tumour size, thus its level may not positively indicate the stage of the tumour. 4) As has been previously reported, other physiologic and pathologic condition, i.e., pregnancy, RA and cardiovascular diseases can also cause the induction the circulating level of VEGF [26,27].

## Competing interests

The author(s) declare that they have no competing interest.

## Authors' contributions

RC designed and sought funding for the study, initiated coordination, performed statistical analysis and drafted the manuscript. OW recruited healthy volunteers and performed the ELISA and western blot analysis. NL recruited cancer patients and carried out the pathological feature examination. UV participated in design of the study and coordination. All authors read and approved the final manuscript.

## Pre-publication history

The pre-publication history for this paper can be accessed here:


